# Where is the limit for overtime? Impacts of overtime on employees’ mental health and potential solutions: A qualitative study in China

**DOI:** 10.3389/fpsyg.2022.976723

**Published:** 2022-12-19

**Authors:** Jiaoyang Yu, Stavroula Leka

**Affiliations:** ^1^School of Education, Taiyuan Normal University, Jinzhong, China; ^2^Business School, University College Cork, Cork, Ireland; ^3^School of Medicine, University of Nottingham, Nottingham, United Kingdom

**Keywords:** voluntary and involuntary overtime, reward, worktime control, mental health, overtime reasons

## Abstract

Although Labor Law of the People’s Republic of China stipulates the overtime system, overtime is still widespread in the IT industry. Through qualitative interviews, we explored the impact of overtime on employees’ mental health and life. The current study identified four main themes, namely overtime reasons, outcomes of overtime, influential factors and solutions to overtime challenges. Besides work-related reasons and cultural influence, it was found that employees may work overtime due to personal reasons, such as capability and personal qualities; the most frequent impacts reported were fatigue, depression, stress and interference with life; three predominant influential factors were timing of overtime, control and rewards; and overtime conditions could be improved in practical and emotional ways. The study’s findings highlight the importance of the design of flexible working time arrangements for avoiding fatigue and improving employees’ work-life balance, enhancement of schedule arrangement for avoiding daily work interruption and last-minute tasks, and optimization of rewarding systems for avoiding complaints and facilitating voluntary overtime. Results suggest that mental health and work-life balance can be promoted by organizational initiatives focusing on minimizing excessive job demands, increasing psychosocial resources, supporting boundary management, and enhancing perceived flexibility.

## Introduction

In recent years, the IT industry has developed rapidly, especially in the field of the Internet. To maintain pace with the competition and to maximize profit, enterprises often need to greatly reduce labor costs and increase the utilization of existing staff and their productivity. Consequently, regular working hours are not enough to complete the required tasks at work. Overtime is becoming more and more prevalent and a sensitive issue.

It is important to reduce and avoid unnecessary overtime work. One obvious reason of working overtime is too much work. The employee’s supervisor or manager and the employee should work together on analyzing and finding out what tasks cause overtime working and redistributing tasks or adding resources. Levenson (2017) stated that too many meetings induce regular work hours not enough to get the job done. While, Watanabe and Yamauchi (2018) indicated that to reduce overtime, it is essential to identify the overtime reasons and tailor relevant measures.

There are three main reasons for working overtime, namely work-related reasons, organizational culture reasons and personal reasons. One of the obvious reasons is that of job demands. Overtime is likely to occur in high demand situations, especially in terms of workload and work pace, can be unmanageable and employees need to work overtime to meet their work obligations (Van der Hulst et al., 2006; Wanninayake et al., 2022). Job demands include extrinsic job demands and intrinsic motivation to meet these demands (Bakker and Demerouti, 2007). According to the Job Demand Control model, job demands could be the predominant determinant of overtime, involving amount and pace of work and work complexity (Karasek, 1979). Work characteristics have been shown associations with overtime hours as employees choose to work overtime to address job demands (Schleupner and Kühnel, 2021). Workload imbalance is one of the major reasons of overtime. Employees may also work overtime to complete the urgent work at hand, especially for new employees (Jeunet and Orm, 2020).

Furthermore, lack of adequate staff can also lead employees to work overtime (Emami et al., 2022). Overtime is frequently used as a major management tool to meet staffing needs due to employee shortages in various settings (Olds and Clarke, 2010; Tanaka et al., 2011). Employees may work overtime as they perceive this to be related to their job security. Especially in the current pandemic situation, the economy is sluggish, and employees are working overtime to keep their jobs (Wang et al., 2019). Lack of sufficient skills and on the job training can lead to overtime work as employees need more time to familiarize themselves with work systems and tasks in order to be able to meet their obligations (Lira and Edwards, 2022). Especially the technology of IT industry is developing rapidly, and employees need to keep learning to keep pace with the times. Coulshed et al. (2022) found that doctors-in-training reported working more unrostered than rostered overtime.

Organizational culture may lead to overtime work, especially when leaders consistently work overtime themselves, acting as role models of appropriate behavior within the organization. China is a collectivist culture context, in which personal traits which can assist collective unified goals are valued, such as individual sacrifice for group goal achievement and contributions toward maintaining harmonious inter-group relationships and perceiving the employer or manager as a power of authority (Kang et al., 2017). In the collectivist context, the employer expects their employees to consider themselves as part of the group. The group goal is much more important than personal interests, so if personal goals do not coincide with group goals, employees are expected to abandon their own needs at certain times to pursue the common goal (Mingyan, 2012). Conformity may be a factor affecting involuntary overtime (Hackman, 1992). Employees have been found to work overtime to help their colleagues to complete group tasks or they work overtime because their manager was still in the office. Therefore, organizations should take the cultural context into account when developing policies to improve overtime working conditions.

In terms of the personal reasons, employees may work overtime motivated by extrinsic or intrinsic motivation. They might perceive this to be related to their promotion prospects or for self-development or they do so to access overtime pay where available (Tucker and Rutherford, 2005; Watanabe and Yamauchi, 2018). Employees who have high sense of responsibility or are imposed on with increased responsibility usually work overtime. Some employees love their company and their jobs, and their sense of passion inspires them to work more (Liu, 2022). Motivations for overtime work are complicated. However, there is a lack of adequate research on individual causes.

Additionally, the development speed of the IT industry is relatively fast, and companies in the industry often need to race against time to meet their targets. The speed of hot spot switching in the industry is relatively fast, and users have higher demands in terms of their experience of internet products, which will prompt IT enterprises to speed up work pace to cope with constant changes in the market and the fast product development cycle (Hang, 2021; Liu, 2022). The first aim of this qualitative study is to identify reasons for overtime work in the Chinese IT industry. The overtime reasons identified can help with tailoring targeted measures from personal and organizational perspectives.

In addition, the impacts of overtime on employees are complex. Previous research has widely investigated the influences of overtime on employees in various aspects, such as mental and physical health (Furihata et al., 2021; Inoue et al., 2021), work-life balance (Fontinha et al., 2019), and job-related outcomes (Okazaki et al., 2019). However, the results of previous studies are not always consistent. A study with a 24-year follow-up did not find any association between overtime work and incidence of depressive disorders (Michelsen and Bildt, 2003). Van Der Hulst and Geurts (2001) failed to find a significant association between psychosomatic health complaints and moderate overtime. Otterbach et al. (2016) claimed that whether the overtime hours were the workers’ preference is much more important for workers’ well-being. The relationship between overtime and mental health does not seem to be simple and straightforward; it seems to rely on the psychosocial characteristics of the work, such as job demands, job variety, job control, and social support (Beckers et al., 2008). Therefore, the second aim of this study was to explore the predominant influences of overtime on IT employees’ mental health and life.

Furthermore, more and more research has started to explore potential influential factors and the solutions that may be able to reduce the adverse influences of overtime on employees (Brokmeier et al., 2022). High (psychological, physical, and emotional) efforts invested but low rewards received is considered as imbalanced, which would induce increased psychosomatic health complaints, physical health symptoms (Verpeléti et al., 2022) and job burnout (Guo et al., 2022). The balancing of potential rewards versus effort investment are central to the phenomenon of mental fatigue (Boksem and Tops, 2008).

The predictive power of the Effort-Reward Imbalance (ERI) model has been substantiated in numerous studies examining the effect of overtime on various outcomes, including depression (Kikuchi et al., 2010; Tsutsumi et al., 2012), anxiety (Calnan et al., 2000), stress (Chin et al., 2022), fatigue (Liu et al., 2020), work-life conflict (Michelsen and Bildt, 2003; Jerg-Bretzke et al., 2020), job burnout (Soomro et al., 2021) and turnover intention (Soomro et al., 2021). Based on the theoretical foundation of the ERI model (Siegrist, 1996, 1998, 2002), the third aim of this study is to explore employees’ attitudes toward rewards and its relevant influence on employees’ mental health and emotional reactions.

In addition to the ERI model, Ala-Mursula et al. (2002) worktime control (WTC) model, highlights that the control over worktime schedule that participants are able to exercise may play a distinct role in overtime. Żołnierczyk-Zreda et al. (2012) reported that employees working long hours but having high WTC suffered decreased mental health problems such as somatic complaints and anxiety, than employees with low work time control. Tucker et al. (2015) demonstrated that low work time control is significantly correlated with greater stress. Another psychosocial factor, voluntary or involuntary overtime, may also exert distinct effects on employees. Even though Shin et al. (2020) found that voluntary overtime was related to nurses’ occupational injuries, Watanabe and Yamauchi (2018) indicated that nurses who engaged in voluntary overtime reported fewer negative emotions than employees who were forced to work overtime visibly or invisibly.

However, most research to date has studied overtime from a quantitative perspective. Research has rarely focused on employees in the Chinese IT industry specifically during the COVID-19 pandemic. This research qualitatively explored the psychosocial working conditions related to overtime. The study explored how IT overtime employees feel from their point of view. Further, this research aimed to explore the ERI model’s applicability in overtime situations and theoretically enrich the fundamental factors of voluntary and involuntary overtime while highlighting that there is still much room for improvement in the protection of overtime employees.

## Materials and methods

### Procedure

To obtain an organization’s permission to conduct the research, the researcher contacted five targeted companies’ managers in the IT industry *via* email who were informed of the research, the researcher’s background, and the reasons why they were contacted. These organizations are distributed in different areas of China. They were China’s top 100 listed IT companies in the last 3 years, engaging in software and services, computer, communication, electronics manufacturing, internet and so on. Approval of investigation was obtained from three managers and a link of the introduction to this research was forwarded to employees by the department of human resources, inviting them to voluntarily participate in the study and informing them that the survey was anonymous and confidential with no personal information to be identified. Ethical approval was obtained from the Ethics Committee of the Division of Psychiatry and Applied Psychology of the University of Nottingham.

The in-depth interview guides involving Chinese participants in the IT industry took a semi-structured format. The current qualitative study focuses on the analysis of the predominant impacts of overtime on employees in the Chinese IT industry relating to mental health, daily life and job-related outcomes as well as the exploration of potential factors moderating these impacts. The interview guides were designed to collect the relevant information for the purpose of the study. The interview questions were designed based on the combination of extensive review of the literature and the purposes of the study and were organized into six sections as follows:

(1) Demographics

Demographic information was asked of participants at the beginning of the interview. It aimed to gather, on the one hand, personal information, including gender, age, education level, marital status and whether or not participants had dependents to take care of; on the other hand, job information was collected, including income and overtime hours on paid and unpaid overtime work. Asking demographic questions at the beginning of the interview was considered as an “ice-breaking” technique. It has been demonstrated to make the interviewees feel relaxed and be beneficial in establishing trust as a good starting point (Shaughnessy et al., 2015).

(2) General information about overtime

This section generally explored the information about overtime in the participants’ current job through the question, “Can you tell me something about overtime in your current job?” DiCicco-Bloom and Crabtree (2006) indicated that the first question should be broad, open-ended and non-leading as the first goal of the interview was to get the interviewee talking. Therefore, this section aimed at enabling interviewees to freely talk about issues surrounding the topic of overtime.

(3) Feelings about overtime

This section focused on how the interviewee felt during or after overtime work. The first aim of the in-depth interview was to investigate the predominant mental health issues among overtime employees in the Chinese IT industry. An example question was: “Can you talk about how you feel during or after overtime work?” Previous studies suggest a correlation between overtime and mental health issues. For instance, Kuroda and Yamamoto (2016) showed that long working hours contributed significantly to deteriorations in workers’ mental health. Workers’ mental health was notably damaged if they worked more than 50 h per week.

(4) Influences on life

This section further explored influences of overtime on employees’ life. Overtime work induces work-life imbalance directly or indirectly, such as sleep disturbance (Virtanen et al., 2009), less leisure time and work–family conflict (Yu and Leka, 2022). For overtime workers, especially for those with child-care or elder-care responsibilities, it has become more difficult to deal with and balance the demands of work and home (Hill et al., 2001). This section sought to explore Chinese IT employees’ experience of overtime in their life. An example question was: “Can you tell me something about the effect of overtime work on your life?”

(5) Opinion on rewards

This section was designed to investigate overtime employees’ opinion toward rewards at work. Issues explored included whether overtime work is compensated by extra rewards, whether they are satisfied by any such rewards, whether they think their effort and reward are balanced and what compensation they would like for overtime work. Working conditions are considered as important factors which are likely to influence the impact of overtime on workers. Whether effort invested and rewards received in a job are perceived balanced or not by overtime workers is associated with reactions such as aversion or willingness to work overtime.

(6) Overall attitudes

The aim of the last section was to let the participants summarize their attitudes toward overtime work and add anything they wished and was not asked in previous questions. The purpose was to allow space for discussing benefits or good feelings associated with overtime work. An example question was: “Is there anything else you would like to add about overtime or related issues?” Therefore, this section enabled participants to comprehensively express their attitudes or opinions and avoided biased understanding or interpretation of findings.

Prior to the main interviewing sessions, the interview guides were piloted on one female and one male who worked overtime in the previous 6 months. This enabled the researcher to grasp how participants responded, how long the interviews would take, how understandable the questions were, and whether any pertinent issues arising could be integrated into the interview questions. The pilot interviews provided the researcher with valuable experience and basic understanding of the topic before the main data collection (Barriball and While, 1994). They enabled the researcher to identify if there were any limitations within the interview design which could be modified before the interviews taking place. It is distinctly helpful to pilot the interview questions and adjust the interview guide accordingly before embarking into formal interviews (Marshall and Rossman, 2016). The pilot interviews were also essential in determining how the interview was to be conducted and estimating the approximate time per interview needed (Kvale, 2008).

A few amendments were made to the original interview guides. Based on the pilot interviews, some questions were re-worded to become as understandable as possible and some prompts were put forward in case participants needed help generating ideas for discussion (e.g., rewards for overtime). The interview would take approximately 25 min, depending on each participant’s response. Formal in-depth interviews were conducted between September 2021 and November 2021. Potential interviewees were approached *via* an email invitation and were given details of the study. They were invited to participate in an online video chat interview with the researcher. Potential interviewees responded by email or telephone and a convenient time for the interviewee was scheduled. Interviews lasted between 25 and 35 min.

The researchers conducted all the interviews *via* Internet chat at a time to suit the interviewee. The Internet interview was appropriate in this study because of the COVID-19 pandemic. Moreover, the researchers were able to conduct the interviews regardless of the location distance. All interviews were tape-recorded. At the beginning of the interviews, interviewees were asked to read a Participant Information Sheet and an “Interview Consent Form” and give their formal agreement to participate in the study. Tape-recording was considered as a preferred method for recording responses as it enabled the researcher to focus on the content and development of the interviews.

Afterwards, interviewees were informed of the interview format and the expected time length of the interview. The research followed the semi-structured interview schedule in the same way for all participants to improve standardization and prevent bias. At the same time, during the whole interview process, the researchers made a conscious attempt not to lead participants in their responses. Clarifications related to research questions were made and any responses that were of interest but not directly associated with the interview questions were duly noted at the end of the interview questions. At the end of the interview, participants were thanked for participating and asked if they had any comments or queries about the interview. They were once more assured of the confidentiality and anonymity about all responses they gave.

In total, twenty-one interviews were conducted. When conducting the nineteenth interview, the researcher found that few new ideas emerged, and therefore, two more interviews were conducted to make sure thematic saturation was achieved. Concerning the number of participants that are typically required to achieve thematic saturation with a homogeneous sample, previous studies have argued that twelve participants are usually sufficient (e.g., Guest et al., 2006; Ando et al., 2014).

The audio recordings were transcribed into text transcripts accurately. After the major process of data analysis, the quotations were translated into English according to Brislin’s (1980) guidelines when writing up qualitative results. The standard “forward-backward” procedure was applied to translate the quotations from Chinese into English. Two independent researchers in the psychological area translated the quotations from transcripts into English. Subsequently they were back translated into Chinese following a care cultural adaptation. The source language version was compared with the back-translated version for linguistic and cultural equivalence and final translations were provided.

### Participants

#### Demographic characteristics

Table 1 shows the personal demographic details of overtime employees in China’s IT industry interviewed in the study. The demographic information collected included gender, age, marital status, dependents information, educational attainments and income. The number of male and female participants was nearly equal. Approximately three quarters of participants were aged 21–30 and held a Master’s degree. 66.7% participants were married, whereas 57.1% did not have dependents been taken care of.

**Table 1:** Demographic characteristics of participants.

	*n* (percentages)
**Gender**	
Male	10 (47.6)
Female	11 (52.4)
**Age**	
21–30	16 (76.2)
31–40	3 (14.3)
41–50	2 (9.5)
**Marital status**	
Married	14 (66.7)
Single	7 (33.3)
**Dependents information**	
Yes	6 (28.6)
No	15 (71.4)
**Educational attainments**	
Bachelor	5 (23.8)
Master	16 (76.2)

Table 2 presents detailed information about the twenty-one interviewees. These are abbreviated from I1 to I21 in the Results section.

**Table 2:** Detailed information of interviewees.

Interviewee	Gender	Age	Marital status	Dependents information	Educational attainments
I1	Female	25	Single	No	Bachelor
I2	Male	26	Married	No	Master
I3	Female	28	Married	No	Master
I4	Male	26	Married	Yes	Master
I5	Female	27	Married	No	Master
I6	Male	38	Married	Yes	Master
I7	Female	25	Single	No	Master
I8	Male	24	Single	No	Bachelor
I9	Female	29	Married	No	Master
I10	Female	45	Married	Yes	Bachelor
I11	Male	27	Single	No	Master
I12	Male	28	Single	No	Master
I13	Female	26	Single	No	Bachelor
I14	Male	32	Married	Yes	Master
I15	Male	27	Married	No	Master
I16	Female	42	Married	Yes	Bachelor
I17	Male	30	Married	No	Master
I18	Female	35	Married	Yes	Master
I19	Female	30	Married	No	Master
I20	Male	29	Single	No	Master
I21	Female	28	Married	No	Master

#### Job characteristics

*Overtime Hours*: The overtime hours of the twenty-one participants in this study ranged from 2 h to 22 h per week. The average overtime hours were about 11 h.

*Income Level*: Table 3 summarizes the income level of the twenty-one participants who had overtime experiences in the previous 6 months in this study. Most of the participants’ income per month was between RMB 3,000–5,000 (Euro 428–714; *n* = 13, 61.9%), three participants between 5,001 and 7,000 (Euro 714–1,000), two participants between 7,001 and 9,000 (Euro 1,000–1,286) and three participants above 9,000 (Euro 1,286).

**Table 3:** Income of participants.

Income level	*n* (Percentages)
3,000–5,000	13 (61.9)
5,001–7,000	3 (14.3)
7,001–9,000	2 (9.5)
Above 9,000	3 (14.3)

### Data analysis

In this qualitative study, the interview data were analyzed by thematic analysis. Braun and Clarke’s (2006) strategies for a thematic analysis were employed, employing six step by step stages: (1) familiarisation with data, (2) generating initial codes, (3) searching for themes, (4) reviewing themes, (5) defining and naming themes, and (6) producing the report. First, the authors read and re-read the interview transcripts to familiarize with data and noted down the initial ideas. Codes were given to the notes across the dataset based on the research questions and existing theory and studies on topics mentioned in the interview objectives. Additional codes were added when the authors immersed in the data and new issues emerged. As this stage progressed, the codes were collated into potential themes or sub-themes and all data were gathered to each potential theme. This resulted in a number of key themes and sub-themes linking the overtime experiences to mental health, conflict with life and job-related outcomes and other related subjects. The initial themes were reviewed and refined to make the themes work in relation to the coded data extracts and the entire dataset. When data met internal homogeneity within themes and external heterogeneity between themes, the authors started another stage, defining and naming themes to identify the essence of each theme. A template was produced which displayed all the themes and subthemes in a hierarchical structure (King, 2004). The template can be found in Table 4.

**Table 4:** Analysis template.

Main themes	Sub-themes	Descriptor
I. Overtime reasons (Why do overtime?)	Work-related reasons	Time node
		Nature of work
		Unexpected/emergent work
		Interruption
	Personal reasons	Capability
		Personal qualities
	Cultural influence	Expectation
		Impression management
II. Outcomes of overtime	Attitudes toward overtime	Positive
		Negative
		Neutral
	Impact on health	Mental health
		Physical health
	Impact on life	Family
		Leisure
		Planning
	Job-related outcomes	Job efficiency
		Work engagement
		Intention to leave
III. Influential factors	Timing of overtime	Frequency
		Intensity
	Control	Time control
		Workload control
		Voluntary or compulsory
	Rewards	With or without Reward
		Effort-reward balance
		Type of reward
		Preference
IV. Solutions to overtime challenges	Practical	Personal
		Organizational
	Emotional	Internal
		External

### Reflexivity

In accordance with the core principles of thematic analysis described above, whereby the researchers’ reflection on their role as an analyst and interpreter is a key part of the research process, reflexivity was undertaken throughout. The direction that the research process took and how the data was interpreted was at least in part, shaped by the personal and academic characteristics of the researcher (Mauthner and Doucet, 2003). The data were coded and themes identified and the analysis then discussed between the researchers. This process allowed for consistency in the method but may have failed to provide multiple perspectives from a variety of people with differing expertise; this could be considered a weakness of the research process, but is all the more reason to offer a reflexive account (Fereday and Muir-Cochrane, 2006). Hämmig et al. (2012) suggested that in order to be reflexive in practice, there are three general areas in which researchers need to demonstrate awareness, and thus should account for the following:

- the personal and professional meanings their topics have for them
- the perspectives and experiences of the persons with whom they wish to do the research
- the audiences to whom the research findings will be directed.

In accordance with Gilgun’s (2008) guidance, the researchers made reflexive notes after each interview and kept a journal throughout the process of qualitative study, including data collection, analysis and interpretation. Relevant reflections are considered to increase awareness of the research process, and a summary of these is presented below.

Participants were selected from three IT companies in China who had overtime experiences in the previous 6 months. Not sharing experiences and standpoint toward overtime with participants puts the author as an “outsider-researcher” at the beginning. With the progress of the interview, the authors may have come to understand, agree with and have empathy with what participants have experienced and their emotional states, which moved the authors toward being an “insider.” Being an “insider” brings both advantages and disadvantages; being an “insider” may arouse sympathy and further stimulate participants to talk more about their overtime experiences; however, this may also lead the research process as participants may focus on expressing what they feel is going to be approved of and/or be emotionally resonant with the researcher (Lewig and Dollard, 2003). The authors’ awareness of the combination of overtime and positive resources that may reduce the negative influences has enabled them to conduct interviews, analyze data and report the findings from a non-judgmental perspective.

Some participants responded with very brief answers when asked about their feelings during or after overtime. After the authors gave examples of the type of experiences, they started to talk more about their own experiences. Conversely, when participants kept talking about what adverse negative influences of overtime brought to them and poured out their complaints, the authors attempted to encourage them to talk from other perspectives. In addition, some participants appeared resistant to talking; when the authors detected this, the authors surmised that participants may have privacy concerns or concerns about the uses of personal data. Therefore, the authors tried their best to ease them and assure them the audio recordings would be deleted after being transcribed in order to help them to relax their guard and increase openness. In addition, interviews were conducted through online video chat, and it has been suggested that in cyberspace there is a tendency to be more open with others, often complete strangers, than in real world communication. Even though the topic of overtime is not a sensitive issue, the online environment may encourage interviewees to give more candid and pertinent responses than in a face-to-face interview (Mark and Smith, 2012; Panatik et al., 2012).

In order to ensure the research questions were appropriate and test the research process, two pilot interviews were conducted, as previously mentioned. Modifications were made and reasonable prompts were put forward according to the feedback of pilot interviews. The authors therefore entered formal interviews with some prior knowledge of participants’ potential feelings and attitudes toward overtime as well as potential psychosocial factors associated with overtime. Not in strict accordance with the interview schedule, the sequence of interview questions was flexible and therefore changed according to the content participants expressed. When the authors captured some important information, they would reflect this back to the participant in order to check understanding and to give participants the opportunity to expand upon or describe further details.

The above reflections are intended to allow the reader some insight into the factors that may have influenced the process of research and analysis, although it should be noted that subjectivity in qualitative research is an accepted and inherent aspect of this methodology, and therefore should not be considered a major weakness in itself (Gummesson, 2000; Alvesson and Sköldberg, 2017).

## Results

There were four main themes identified and in each theme section, sub-themes are presented in conjunction with supporting quotes from the participants. These are detailed next.

### Overtime reasons (why do overtime?)

A key introductory aim of the qualitative research was to ascertain the reasons for participants in the IT industry working overtime. The first theme shows the reasons for working overtime from the sample in the IT industry based in China. These categories were collated by the researchers from the thematic analysis.

#### Work-related reasons

Participants gave various reasons for working overtime from different aspects. The most prevalent of these were work-related reasons, including time node, which refers to a special time period, such as “the end of month” (I1), “when overtime work was frequent” (I3); the nature of work, which means some elements of work may cause overtime, such as “high workload” (I5), “job position requirement” (I6), “work flow” (I9), “work complexity and work schedule” (I15); unexpected/emergent work, which includes “short notice” (I10), “emergent tasks” (I12), “uncertainty at work” (I16); and interruption during the workday, which includes “inspection, reports or unexpected meetings” (I12), “training and disturbance by other things outside the work” (I17).

Regarding the seriousness of the impact of the above situations, high workload usually causes chronic fatigue: “I cannot finish my work every day. I’m really tired” (I5), whereas emergent work and short notice work usually induce anxiety and acute fatigue: “When I work overtime in an emergency, I will be anxious and stay up late. The mind is tense and the body is tired and sometimes I will lose sleep” (I10).

#### Personal reasons

Participants also explained that they engaged in overtime due to personal reasons, such as capability and personal qualities: “When your skills are not good enough for the current task, you need to learn something new and usually have to work overtime” (I4). However, another participant expressed that competent workers usually had more tasks and worked overtime more: “the amount of overtime reflects the effectiveness of a person or the importance of a position. A capable man is always busy… Tasks are not equally distributed… The hours and amount of overtime are related to your ability and importance” (I7). Additionally, some participants mentioned personal qualities as an invisible reason for influencing the decision to work overtime or not: “a person who works overtime usually has a sense of responsibility” (I11).

#### Cultural influence

A country’s traditional culture or organizational culture influences workers’ performance, attitudes and behavior. Chinese workers usually perceive their manager or superior as a person with authority. They behave or perform according to their managers’ expectation. In addition, the group makes workers bond tightly and influence each other. Workers’ overtime is affected not only by their tasks and personal intention but also by the working environment (Mingyan, 2012). Common themes in this study included “manager or leader’s expectation” (I8) for overtime on participants. Also important was participants’ impression management on others: “sometimes we work overtime because the leader is still in the office” (I13) or “at the end of each month, the whole group is working overtime, so sometimes even though your work is complete, you still stay there” (I18).

### Outcomes of overtime

The thematic analysis sought to uncover the impacts of overtime. The following part details the results of the thematic analysis with respect to the subthemes of attitudes toward overtime; impact on health; impact on life; and job-related outcomes, which appeared to be related to the mechanism of overtime discussed above.

#### Attitudes toward overtime

A few participants held a neutral attitude and admitted that overtime was unavoidable, inevitable and necessary at work: “it was common and usual in the work. Moderate and occasional overtime was acceptable” (I14). Different aspects of overtime induced different attitudes. Positive attitudes toward overtime were mainly contributed by factors such as: “the overtime was moderate” (I11); “it could be planned or scheduled in advance” (I15) by participants; and the overtime was appropriately rewarded: “if there is an overtime fee, I am willing to work overtime because every minute you work is rewarded. When you do a thing and get some benefits, you will feel that you are willing to do it” (I18).

On the opposite end, participants held negative attitudes toward overtime, which were “long-term and high-intensity” (I7); temporary or emergent: “temporary and sudden overtime is more annoying. Especially if the day has been arranged, I have to cancel some of my plans which will be disrupted. It is really disgusting” (I2); compulsory: “I do not want to be forced to stay at the office at night and sit there” (I19); “no or less rewarded” (I9); as well as meaningless and unproductive: “I have become more and more resistant to working overtime… I do not want to be forced to stay at the office at night and sit there. The efficiency is quite low” (I19).

#### Impact on health

When asked how they felt during or after overtime work, participants responded in relation to two areas, namely mental health and physical health. The main issues discussed in relation to mental health have been further classified as depression, stress and anxiety. The definitions and symptoms used in determining these constructs are provided in Table 5.

**Table 5:** Evidence-based definitions and symptoms of depression, stress and anxiety.

Constructs	Symptoms
Depression	Feelings of sadness, tearfulness, emptiness or hopelessness (Dekker, 2014)
	Sleep disturbances, including insomnia or sleeping too much (Nutt et al., 2008)
	Tiredness and lack of energy (Braley et al., 2012)
Stress	Environmental and/or internal demands exceed the individual’s resources for managing them (Holroyd and Lazarus, 1982)
	A reaction to a stimulus event (Skinner, 1985)
	Stress can be defined as “an underload or overload of matter, energy or information input to, or output from, a living system” (Steinberg and Ritzmann, 1990, p. 140)
Anxiety	An emotion characterized by feelings of tension, worried thoughts and physical changes like increased blood pressure (Kazdin and Kazdin, 2000)
	A state consisting of psychological and physical symptoms caused by a sense of apprehension at a perceived threat (Akiskal, 1985)

Symptoms of mental ill health, such as depression and anxiety, were reported to be caused by overtime. Although participants did not specifically use the words depression, they did refer to a variety of feelings related to depression including feeling “spiritless” (I20), “inactive” (I20), “unhappy” (I15), “miserable” (I17), “dysphoria” (I11), “irritable” (I9) and so on. For two examples: “if I worked overtime late in the evening, I felt quite tired after going home and did not want to speak. The next day I would be spiritless and became inactive” (I20); and “if there were so many tasks during these days, I felt upset, really upset. It seemed that overtime was never over” (I9).

In addition, participants may feel anxious during the overtime work or after the overtime: “sometimes, for example, there was a case coming in at nine o’clock in the evening, and then you were asked to get it completed by ten o’clock. During that moment you were quite crazy because you were urged to deal with this thing, so at that time you felt very anxious” (I10). Another participant expressed that temporary overtime that was demanded at short notice created a state of anxiety for him: “at the weekends, for example, you were playing outside, but you were afraid of your phone ringing to call you back to the office and work overtime… You would definitely feel more or less anxious” (I6).

Participants experienced aggravating stress or tension during or after the overtime: “even after overtime, you were always thinking how to solve the problem. That pressure was actually big” (I3). The influence was particularly significant when the overtime was emergent: “sudden overtime was also urgent. We worked very late, sometimes to the middle of the night. The manager would also come to the office to discuss countermeasures. So, the pressure was particularly great for this emergency overtime” (I12). or of high intensity: “in the month of May, overtime was very frequent. From the beginning of the month to the twentieth basically, every day I worked late. Then the intensity was relatively high, the pressure would be relatively great” (I14).

With regard to physical health, the two most popular issues were fatigue and sleep problems. The most serious issue was fatigue; nearly all of the participants expressed they felt tired during or after overtime work. Some overtime fatigue was acute, usually caused by high hours overtime in 1 day or in a short time: “tired, indeed I am really tired. Especially sometimes when I have worked overtime until eleven o’clock in the evening, I am really tired” (I16). Another interviewee claimed that “if a case must be completed today or in such a situation, we would stay up very late, sometimes until the early morning. It was particularly tiring” (I4).

For those who worked overtime continuously, they suffered from accumulated and chronic fatigue: “if it is a kind of long-term overtime, you certainly feel, oh, tired. If the recent task is heavy, or the frequency of overtime is high, my brain will be in a state of exhaustion and excitement. You cannot rest well” (I2). Some participants specified that they felt tired when they had “insufficient rest and bad recovery during the overtime period” (I5). Three participants explicitly expressed that they needed a rest to recover from the overtime especially after the long-term or high strength overtime: “after overtime, I was quite tired and wanted to quickly go home to take a rest” (I10).

Another serious matter suffered by participants was sleep difficulties, including insomnia, bad quality of sleep, irregular sleep and less sleep time: “after long hours of overtime work, your brain was still in a state of excitement. In fact, you were unable to fall asleep after going home, but tossed and turned in bed over and over again” (I2). Participants also claimed that overtime increased the risks of other health issues, such as “sickness” (I8), “headache” (I12), “cervical spondylosis” (I3), “lumbar disc herniation” (I17) and “irregular diet” (I19). For an example, “My personal feelings and experience are, if I work overtime 2 days a week for continuous 2 weeks, I will get a cold, definitely get a cold. The body really cannot stand it, not just psychological pressure. Then when thinking I have to work overtime again, I feel a headache and brain swelling. It is not just every day’s fatigue, but health is really impacted” (I8).

#### Impact on life

Three subthemes of overtime influence on life were family, leisure, and planning. The first impact of overtime is on overtime employees’ family, especially for participants with dependents, they had “less time to take care of and accompany child or parents” (I14). Some participants’ families were also affected more or less: “frequent overtime may induce family members’ misunderstanding and conflicts with them” (I18). Similarly, participants may bring the adverse emotions to families after overtime work: “if I worked overtime late one night, I was very tired after going home and did not want to speak. If I was in a bad mood, I would bring it to my family and my tone speaking with my family was not very good” (I16).

Another major impact of overtime on participants’ life was leisure. Participants complained that the overtime reduced their time for “entertainment activities” (I3), “leisure” (I8), “exercising” (I13), relaxing: “the overtime decreases your personal time for rest” (I19);“overtime takes up your time to, for example, go out for dinner, see a movie, or go to the gym” (I4).

Overtime work prevented participants from making a plan for other activities in life. In particular, temporary or emergent overtime disrupted plans already established: “as our job is IT maintenance, for example, when the system fails suddenly, then you are asked to go back urgently to check the data or cause, or you are made to go to an emergency business trip… No matter if it is the weekend or day and night, you are on call. Tickets were booked in the morning and you left in the afternoon. Planned good events were all upset” (I21). Compared to temporary overtime, overtime that can be scheduled in advance to a large extent reduced interruptions: “you originally arranged the weekends for an activity, going out or for other entertainment, but suddenly you are asked to work overtime… Then you have to. Instead, if the overtime work was able to be scheduled, no matter how late I worked on Friday, I can plan for my Saturday and Sunday” (I13).

#### Job-related outcomes

Another issue relating to overtime is job-related outcomes, including job efficiency, work engagement and intention to leave. The overtime work, which is less productive and inefficient, may be caused by high workload, frequent overtime, long-term overtime, compulsory overtime, negative and reluctant overtime: “the temporary overtime which disrupted my plan made me feel resentful and resistant. At least I cannot accept it calmly. The overtime efficiency would not be particularly good” (I20) and “I do not want to be tied to the office every night, sitting there, and the efficiency is pretty low” (I11).

It should be highlighted that employees who decreased their engagement at work because they felt the imbalance between their effort invested and rewards received: “there’s only monetary compensation for dinner after a certain time of day… I do not work overtime now. The imbalance reduced my enthusiasm for work” (I15). Additionally, participants who reduced their work engagement mentioned above expressed the intention to change the current job because of lack of compensation for holiday, no reward for overtime work, no opportunity for development or promotion in their jobs: “I have thought about changing jobs because I heard from someone in another company that they can accumulate overtime hours in exchange for holiday. They can, why cannot we, so I want to change jobs” (I9); “there’s no reward for overtime work and I cannot see any development or promotion in my job, so I want to search for another job if possible” (I12).

### Influential factors

One key purpose of the qualitative interviews was to probe for the possible factors affecting the effects of overtime. Three subthemes of potential moderating perspectives, timing of overtime, control and rewards make up the theme influential factors.

#### Timing of overtime

In this study, participants mentioned that overtime differed in terms of frequency and intensity. Some participants stated that the frequency of overtime was relatively low on usual working days, which causes “less stress, fatigue and work–family conflict,” but at the end of the month or during specific periods, the overtime was frequent: “when it comes to the key time node of the project, such as mid-term inspection or evaluation, continuous weeks of overtime occur,” which is really stressful and tired” (I2). Moreover, when participants mentioned high frequent overtime in a period, they usually specified the overtime was high intensity: “when we were busy, we worked 6 days a week and 11 hours per day… Long-term, continuous overtime is often high intensity” (I7). Some participants specified that sometimes long hours’ overtime on 1 day was also intensified: “once we worked overtime until two o’clock in the morning. I was really exhausted. It was a miserable experience” (I19).

#### Control

Another theme emerged from participants’ responses. Participants considered the control at the work they possessed crucially related to the impact of overtime. Participants believed how much autonomy they have on working time was influential: “it depends on myself when to start and end work during the working day” (I9). Overtime control is also important: “it depends on whether the overtime is under your control or if it is temporary overtime that you cannot control, which I think makes a great difference. It is okay if I can arrange the overtime. If it is always temporary overtime, I will be irritated” (I21). In addition, “sudden and emergent tasks are usually out of control” (I8). In addition, control over the workload may also exert an influence: “when you feel the task is too much, you can talk with your boss that you have too much to do recently and then tasks can be adjusted. You do not have to keep doing it all the time” (I15). On the other hand, a participant expressed less control over the amount of work: “if a task is assigned to you, you have to work overtime and complete your work” (I19).

Moreover, the most frequent issue mentioned was overtime control, focusing on whether it was voluntary or compulsory. Participants gave various factors that may contribute to the decision to undertake voluntary overtime: reasonable and moderate levels of overtime and autonomy on overtime schedule: “if the overtime is reasonable and appropriate, and it can be arranged by myself, it is acceptable” (I11); and appropriate compensation: “if there is overtime pay, I like to work overtime because the time I spend on overtime is rewarded” (I1). In terms of compulsory overtime, participants gave a number of responses about factors causing compulsory overtime, including “temporary urgent increase [in work]” (I8), high workload: “at the end of each month, there are many business cases, so we are busy and overtime work is required” (I19), continuous and high intensity overtime: “I have an aversion to long-term and high-intensity overtime. Even though I am reluctant, I have to do it and have no choice” (I17).

#### Rewards

Understanding rewards relates to with or without rewards, effort-reward balance, type of reward and preference. When asked about the issue of rewards or compensation for overtime, participants firstly expressed whether they received rewards or not. Nearly half participants expressed that they had no or almost no rewards, indicating that no matter how many overtime hours they worked, there were no compensated rewards for the extra time investment. One interesting finding was that two participants specified that they had rewards previously, but now they did not have any: “overtime rewards are based on pay grades. If your salary is at a relatively low level, in order to compensate your entire income monthly, there is overtime pay. But if your salary reaches a certain level, there is no overtime pay. So I had overtime rewards previously, but now I do not” (I10). In addition, some participants expressed that they had specific rewards for overtime whereas some participants indicated that they did not have specific rewards for overtime hours, but they received rewards after the tasks were completed.: “if you work overtime and get some work achievements, there will be some overall reward for your performance at the end of each month or year” (I9).

When asked if participants felt their effort and rewards were balanced, participants who worked overtime but received no or little rewards, felt unrewarded, imbalanced and unsatisfied, especially for participants who worked overtime regularly and long term: “nominally the reward will be reflected a little in the performance. But it still does not meet our expectations, or the reward does not obviously reflect our effort. The overtime payment is terrible” (I3). Participants felt exacerbated dissatisfaction when the overtime was compensated with imbalanced rewards and it was compulsory: “now I’m more and more averse to overtime. I do not want to be forced to sit in the office to work overtime after work in the evening and there are limited rewards for overtime work” (I5).

The participants who received rewards for overtime based on company regulation thought it was acceptable and moderately balanced: “Overtime pay on working days is 1.5 times as much as usual, 2 times on Saturday and Sunday and 3 times on statutory holidays. It is a relatively standard compensation” (I11). Some participants expressed even though there were no specific rewards for overtime, they felt their effort invested and rewards received at work were balanced to a large extent: “when you complete a task, it has a bonus. It is proportional with the task: more tasks, more bonus and fewer tasks, less bonus. Our work is rewarded in that way, mainly by performance assessment” (I13).

In terms of reward type, participants received money or some compensation for overtime, including “supplementary vacation” (I16), “overtime dinner and taxi fee” (I6); and some participants referred to the possible “development opportunities at work” (I3), although this type of reward was less certain.

When asked what rewards or compensation participants would like better for overtime, money compensation is important in the first place. Participants who did not receive financial rewards for overtime felt more strongly that monetary rewards should be given. However, financial rewards were not the preferred desire of overtime compensation for all participants. A small number of participants preferred compensation in time: “as far as possible I want time compensation for the hours I spent on overtime. The compensation for money is secondary” (I18).

In addition, apart from the rewards of money and time, some participants hoped to get some emotional support from their manager or boss, including verbal rewards and spiritual encouragement and care: “after overtime, you certainly want to get approval or something like that. The boss knows that you are working overtime and you are working hard. The boss could give some acknowledgement to you on “*wechat”* [a social software in China] or the next day ask you about yesterday’s overtime work” (I20). A few participants also had a preference for development in their career, including “promotion” (I6), “skill improvement” (I11) and “good learning opportunities” (I4). What should be noted is that one participant stressed that no matter what rewards were compensated for overtime, she did not want to work overtime: “overtime for me, any compensation is not worth it because I will get sick. Even if you give me money, in fact, my body cannot recover” (I10).

### Solutions to overtime challenges

The last theme includes potential opportunities to improve existing practices of overtime risk management and improve the conditions of overtime and reduce the negative influence of overtime, which consist of personal solutions and organizational solutions.

#### Personal

Practical ways to reduce the adverse impacts of overtime could combine both personal and organizational aspects. It was suggested advocating efficient work and encouraging employees to complete their own tasks within working hours: “one requirement for myself is trying to improve work efficiency to finish work in a limited time, so that I can balance my work and life, rest time and entertainment time” (I2). Employees should seek effective solutions: “When the individual ability is insufficient, the work quality and progress may be affected by individual completion. At this time, it is necessary to report to the leader and ask other colleagues for support to avoid delaying the work process” (I15). The other way was to improve emotional support from personal internal adjustment: “there is no need to be always in the mood of resentment about overtime. After all, the nature of work is like this” (I6); and gaining more external support: “sometimes it is helpful that the superior manager comes to look around and motivate you during overtime” (I21).

#### Organizational

Organizations should formulate legal and effective enterprise rules and regulations, correctly calculate workers’ overtime pay, “it is best for the company to provide some overtime rewards to employees. They should not let this become one incentive to induce employees to complain or even leave the company” (I3); “If the overtime work is really emergent and necessary, nevertheless the company should improve the compensation for overtime” (I17). The overtime work performed due to personal leave (personal affairs, sick leave, study leave, marriage leave, etc.) and unfinished work within the working hours can be done in the form of compensatory time off: “it is inevitable for everyone to take time off and the company should allow some flexibility” (I12).

## Discussion

### Comparison to previous research

In this research, some findings are consistent with previous studies whilst some new findings are generated. The following section compares the findings within the four main themes with previous research and outlines areas of commonality and highlights where this research offers new insights.

Firstly, in terms of overtime reasons, namely why employees do overtime, investigation of the results in this research revealed that work-related reasons, personal reasons and cultural influence were the major causes making employees to work overtime. Two of these, work-related reasons and cultural influence are consistent with previous research findings; for instance, Houdmont et al. (2011) identified four reasons for overtime: job demands, work culture, intrinsic motivation to work overtime, and anticipated rewards for overtime; similarly, Van der Hulst et al. (2006) and Riedy et al. (2021) indicated that work characteristics had strong impact on working overtime. The current study also found that work-related reasons seemed to be the most prevalent reason, including time node, nature of work, unexpected/emergent work and interruption, in which nature of work and unexpected/emergent work were especially significant influence factors inducing overtime work. This is consistent with the findings of Tsutsumi et al. (2012) who indicated that job demands such as high workload contributed significantly to overtime work as well as Houdmont et al. (2011) who found that job demands were the most influential predictor for overtime hours.

In the current study, cultural influence was also found to exert an underlying influence on employees leading to overtime work in the workplace. For instance, participants choose to work overtime in order to cater to management expectations and make a good impression. Cultural influence had been demonstrated in the previous research based in both individualist and collectivist cultural contexts. Goldenhar et al. (2003) found that culture influenced the decision on working overtime in the construction industry of United States. Workers usually say yes when the manager asks them to work overtime and if found to be refusing it many times, they would find their career impeded. This demonstrates the cultural norm surrounding what it takes to forge a good career in an individualist society, and whilst the pressure to undertake overtime in a collectivist society has a similar outcome, the reasons are somewhat different. Triandis (1995) indicated that in a collectivism society, the group bound members tightly and made them influence each other at work such that they would work overtime to complete the group goal. Further, Houdmont et al. (2011) found that Chinese office workers were influenced by the overtime work culture in which employees usually see themselves as parts of the organization and expect to take on the duties imposed by it, which is in line with the findings in the current study; here, participants claimed that they still stayed in the office and worked overtime after work despite that they had completed their own work but in order to help other colleagues in the same group. It is worthwhile noting that the personal reasons for overtime found in this research (namely capability and personal qualities), to the authors’ knowledge, have rarely been investigated in preceding studies.

Secondly, the major outcomes caused by overtime were identified, including attitudes toward overtime, impact on health, life and job-related outcomes that align with previous findings. Overtime participants held positive attitudes or at least they felt overtime was acceptable if the overtime was reasonable and moderate, was able to be scheduled in advance by themselves and was rewarded appropriately. This is entirely in keeping with previous research. For instance, Tucker and Rutherford (2005) indicated that workers viewed overtime positively because of the financial rewards, meanwhile on the other hand, negative attitudes have been associated with high intensity (Wanninayake et al., 2022), effort-reward imbalance (Aronsson et al., 2021) and less control (Yu and Leka, 2022). Mental and physical health problems arise due to overtime that was supported by previous findings. This study found that overtime work was associated with depression which was consistent with Ishikawa’s (2022) study. Zadow et al. (2021) indicated that overtime work acted as a predictor of major depressive episode. This study also found overtime was a risk factor causing stress (Goldenhar et al., 2003).

In terms of physical health, the current research found overtime work was related to increased fatigue, bad recovery and poor sleep that were consistent with previous research (Hagan-Haynes et al., 2021). Van der Hulst et al. (2006) indicated that overtime employees need recovery especially in high strain jobs (high demand, low control). Goldenhar et al. (2003) claimed the extended overtime work could disturb workers’ sleep schedule and was negatively associated with their sleep quality. Virtanen et al. (2009) found long working hours shortened sleeping time and increased difficulty in falling asleep. Additionally, several physical health problems identified in this study, including cold, headache, cervical spondylosis and lumbar disc herniation, were identified in previous findings (Caruso et al., 2004; Kawada and Ooya, 2005).

Regarding effects on life, overtime work was found to induce work–family conflict, leisure time deprivation and interruption toward plans already made, which were consistent with the literature reviewed. For instance, Goldenhar et al. (2003) found the stress of overtime brought to lives outside of work. Long working hours is associated with work-family imbalance, including taking care of children and parents, doing housework and communicating with partners (Grzywacz and Marks, 2000). Other studies have found that leisure time decreased due to working overtime (Grzywacz and Marks, 2000; Hämmig and Bauer, 2009). Furthermore, this research highlighted the interruptive influence of overtime on worker’s schedule outside the work. In particular, emergent or temporary overtime exerts a much more negative impact. Siegrist (2002) claimed that in the software industry, overtime planning was extremely poor caused by “estimate inaccuracy and time-to-market pressure,” leading to detrimental influence on workers’ quality of lives. On the contrary, overtime that can be scheduled in advance by workers causes less interruption. In terms of this finding in current research, namely the workers’ autonomy on overtime schedule, to the authors’ knowledge, there is still a shortage of previous studies specifically investigating this.

Job-related outcomes in terms of job inefficiency and turnover intention found in this research are supported by previous literature. Shimizu et al. (2004) reported that low productivity was closely related to overtime working, whilst Hoseini et al. (2021) found that overtime increased workers’ turnover intention. Although previous studies have indicated a relationship between overtime and less organizational commitment and low job performance (e.g., Adler et al., 2006), few studies have explored the impact on work engagement. In demonstrating that overtime work, especially compulsory and inappropriately rewarded overtime, decreased workers’ enthusiasm to engage in work, the current research therefore adds to the body of knowledge in this topic area.

The findings of the third theme suggested the potential influencing factors that may aggravate or buffer the impacts of overtime. The influence of frequency and intensity of overtime, (here, the first sub-theme of influential factors), is a finding that aligns with previous studies. In the present study, participants considered occasional and moderate overtime to be reasonable and acceptable at work, whilst, long term and frequent overtime was objectionable. In terms of previous research, there are consistent findings that demonstrate the detrimental impact of high intensity overtime, whereas the adverse impact of moderate overtime on health and well-being has not been consistently demonstrated (Geurts and Sonnentag, 2006). Taris et al. (2011) stated that working a moderate amount of overtime did not usually cause elevated risks of health whereas these would worsen with increasing overtime. Dahlgren et al. (2006) concluded that 1 week of overtime work with a moderate workload created no significant effects on physiological stress; however, sleep was adversely influenced, with insufficient sleep during overtime work and increased problems of fatigue and sleepiness. In addition, the present study highlighted frequent overtime may bring about long hours overtime and high intensity, further inducing chronic fatigue. High intensity overtime is not merely caused by weekly or monthly long hours of overtime. One day’s long hour working can also induce high intensity overtime, such as overtime working until mid-night that would induce acute fatigue. High frequency and high intensity result in long hours’ overtime that would be related to chronic fatigue.

This research also identified two psychosocial factors that might exert distinct influences on overtime, namely control and rewards. Regarding control, several findings of the present research tie in well with results of previous studies. Regarding the control that participants can exert, the most predominant were worktime control and workload control, confirming the results of previous studies that suggested that job control is a potential factor moderating overtime’s influences (Aronsson et al., 2021). More precisely, this study supported the notion of flextime, namely when to start and end the time of workday, in Ala-Mursula et al. (2002) WTC model; the current research also specified the control over overtime schedule that how much control participants were able to dominate may play distinct role in overtime. For instance, overtime work which can be arranged and planned in advance by participants, exerts less adverse impact, whereas temporary or urgent overtime induces much more serious influence. It seemed that the more autonomy the participant has, the less adverse impacts the overtime causes. Tucker and Rutherford’s (2005) study claimed that the impact of overtime appeared to be contingent on the amount of control that workers have over the overtime hours and when to work overtime. However, the qualitative results in the present research did not identify the other sub-dimensions of WTC in previous studies, such as break control and the distribution of workdays over the workweek (Geurts et al., 2009), which may be because these aspects were not available to these participants or participants did not mention them as the interview schedule did not focus on worktime control.

In addition, this study highlighted that voluntary or compulsory overtime was one specific factor inducing distinct influences that was consistent with limited previous studies. This research suggested that compared to voluntary overtime, compulsory overtime caused much more serious negative feelings, such as resentment, stress, aversion, anxiety and fatigue. Ota et al. (2005) indicated that voluntary and compulsory are two opposite poles of overtime control; involuntary overtime workers suffered from higher fatigue and lower satisfaction and rewards may partly reduce the adverse effects of compulsory overtime. The current research found that three factors contributed to a willingness to work voluntary overtime: these were autonomy on overtime schedule, reasonable and moderate overtime, and appropriate compensation. Compulsory overtime was caused by urgent tasks that were out of participants’ control, high workload, continuous and high intensity overtime, and unsatisfying rewards. Previous research (Watanabe and Yamauchi, 2016) found two reasons for engaging in voluntary overtime: intrinsic motivation and extrinsic motivation; whilst two reasons for having to undertake involuntary overtime focused on workload and conformity, which was consistent with this present study’s findings that rewards were likely to stimulate employees to engage in voluntary overtime; constant and high intensity overtime and cultural influences seemed to make employees engage in involuntary overtime.

In terms of rewards, several participants in this study received specific compensation for overtime which was investigated in previous studies (e.g., Ota et al., 2005; Sasaki et al., 2007). The present study found that even though some participants received compensation for overtime, they felt that the compensation was not enough for their extra effort invested in the overtime work. There were also some situations in which there may be no specific compensation for overtime, where instead the overall rewards were given based on job output or performance. On the opposite end, even though some participants did not receive specific compensation for overtime, they felt satisfied with the overall reward at work compared to their effort invested including overtime work.

Therefore, considering these, in addition to measure compensation for overtime, one of the most influential models in overtime research is Siegrist’s (1996) Effort-Reward Imbalance model (ERI) and to a large extent the findings of the present study support the large body of research that previously endorses this model. Briefly, the model investigates the balance between effort invested and rewards received at work, which is classified into three levels, effort more than rewards, effort balanced with rewards and effort less than rewards, which was widely used and further investigated in previous studies (Van Der Hulst and Geurts, 2001; Li et al., 2005).

This model was relevant to the current study as ERI especially causes stress and dissatisfaction in the overtime situation. This research suggests that whether employees could feel balance between effort invested and rewards received at work makes a big difference in overtime’s effects. When participants felt imbalanced, adverse effects were produced, including bad emotions, decreased motivations, less work engagement and increased turnover intention; on the other hand, when participants perceived balance between effort and reward, they were less against overtime and performed overtime work with less negative emotions; these findings were consistent with previous research, indicating that effort-reward imbalance induced negative outcomes on mental health (Griep et al., 2021) and job-related outcomes (Wolter et al., 2021). Bell et al. (2000) found that workers who were remunerated higher wages invested more effort and hours above the paid hours at work, whereas overtime without rewards was negatively related to work productivity.

In particular, the current research highlighted that the negative impact of effort-reward imbalance was increased accompanied by involuntary overtime working, which is in keeping with the research by Van Der Hulst and Geurts (2001), which found that the adverse impact of low job rewards was exacerbated by the compulsory overtime work applied to workers. In terms of the type of rewards, Ota et al. (2005) measured rewards for overtime including money and time. The current study identified financial compensation, time compensation and promotion or learning opportunities in career. Participants in the present study held different preferences toward rewards; to the authors’ knowledge, this preference for rewards was rarely explored in previous studies.

Finally, this study found several solutions to overtime challenges, although this topic was not widely referred to by participants. There were two possible ways to improve overtime conditions from practical and emotional perspectives. In terms of practical ways, improvements can be achieved from both personal and organizational aspects. “Organizational aspects” supports findings in previous studies investigating solutions to decrease adverse impacts of overtime, mainly focusing on worktime autonomy arrangement (Geurts et al., 2009; Yu and Leka, 2022) and improvement of compensation for overtime (Westover and Taylor, 2010; Huang, 2016). However, “personal aspects” has been rarely investigated in previous studies. In terms of emotional ways, this study indicates solutions relating to internal self-adjustment and external social support. This finding supports previous studies investigating the general and positive impact of social support in working conditions (Pomaki and Maes, 2002; Moen et al., 2011), however self-adjustment lacks investigation in previous studies. Solutions in personal ways need more investigation.

Overall, this study found that personal reasons inducing overtime seemed to cause fewer negative attitudes toward overtime, such as lack of skills and interruption by personal issues. This study also suggested that autonomy over worktime and overtime and appropriate rewards would arouse voluntary overtime and further induce fewer negative emotions and impacts. On the opposite end, high intensity and out of control overtime, and unsatisfying rewards would cause involuntary overtime and further lead to elevated negative influences.

### Strengths and limitations

This qualitative study provides detailed information about overtime experiences among employees who worked in the IT industry in China; even though overtime working is prevalent in the IT industry, it has been rarely investigated in previous studies; furthermore, research on overtime in China is relatively rare with most overtime research focusing on Western countries.

This study provides a comprehensive picture of overtime influences, including health, life and job-related outcomes. This qualitative research will enable more effective triangulation of previous findings using quantitative methods. This study explores autonomy over overtime schedule, with the findings on the attitudes and impacts of planned versus unplanned/emergency overtime contributing new insights to the field of overtime research.

However, a relatively small number of participants were interviewed, which could be considered a weakness of this research. There may also have been some selection bias as employees who responded to invitation letters may have had different characteristics and experience compared to those who did not respond or declined to take part. For example, most interviewees did not have dependents which may differ in a wider sample. In addition, the designation of participants’ experiences as “mental health problems” was achieved by the matching of phenomena that participants described and academic definitions of specifically identifiable mental health issues. However, it is possible to argue that these categorizations may lack an element of precision. For instance, it is hard to distinguish or identify clearly whether fatigue is mental fatigue or physical fatigue in terms of participants’ relevant descriptions. In addition, the themes were generated and identified from the interview data. Further research can investigate further possible additional issues of relevance to employee health and well-being as a result of overtime work.

### New findings and research implications

Firstly, this current study may have made some contributions to the academic fields. Findings in the current research suggest that current Western theory and evidence might also apply to some extent in the collectivist cultural context, but this suggestion needs more research to strengthen its validity. This study indicated overtime reasons: work-related reasons and cultural influence found in this study were investigated in previous studies and were consistent with preceding findings, whereas the current study found that employees may work overtime due to personal reasons, such as capability and personal qualities, which was rarely identified in previous research. Personal reasons seem to be a new finding that needs further exploration. This new finding suggests future studies should widely explore issues of overtime brought about by personal reasons, as this may lead to employees’ distinct attitudes and emotions toward overtime and further cause different levels of impacts on employees. For instance, if there is interruption during the work caused by personal issues outside the work, employees may tend to work overtime voluntarily to complete the work on that day. If employees’ skills are not enough for current tasks, they may work overtime to improve their capability and get the work done. In addition, employees who have high sense of responsibility toward their organization may engage in overtime working.

Secondly, some recommendations have been put forward in terms of actions taken by the organization that might improve the situation surrounding overtime and lessen the negative impact of overtime. These include encouraging the organization to improve strategic planning to reduce the need for last-minute, unplanned overtime. Employees may work overtime due to a lack of useful competences at work, meaning that work cannot be completed in the allocated time; in this case, the organization should provide employees with training opportunities to improve specific competencies or skills required at work. Employees may work overtime due to the interruption by personal issues during the workday, and in such cases worktime flexibility would be beneficial to employees that they can adjust the work time according to their present situation and prevent overtime work. Further investigation about the personal reasons for overtime is needed to understand overtime reasons comprehensively.

In terms of control, although workload control was not mentioned as frequently as worktime control in participants’ responses, it was considered as one potential factor that may cause overtime and result in compulsory overtime. It is recommended that future research investigates job-related control in various aspects. Furthermore, this study indicated that rewards were likely to reduce overtime employees’ complaints and negative emotions. In addition, participants held different preferences toward rewards that suggest organizations should improve their rewarding system to reward employees appropriately and base rewards on employees’ preferences to maximize the positive effects of rewards. This study adds up to Ala-Mursula et al. (2002) worktime control (WTC) model, specifying that control over overtime schedule, how much control participants were able to exercise, may play distinct roles in overtime, demonstrates the ERI model’s applicability in overtime situations and theoretically enriches the fundamental factors of voluntary and involuntary overtime.

Thirdly, this qualitative study provided some guidance for the next phase of future research, namely the influences of involuntary and voluntary overtime. This study identified some of the factors that resulted in overtime being undertaken on a voluntary versus involuntary basis; this is important because the voluntary versus involuntary nature of overtime further results in different outcomes. Findings demonstrate that the outcomes of working overtime on a voluntary basis are much more positive than where overtime is worked on an involuntary basis. This is a new and important finding within the current study as it shows the need for employers to attend to the factors that promote voluntary overtime working, rather than to rely on making overtime compulsory for employees. Employers should be encouraged to plan strategically and promote autonomy and flexibility among their workforce, with a view to achieving the positive organizational and individual outcomes that are associated with voluntary overtime.

### Conclusion

This paper presented an exploration of overtime employees’ experiences of overtime and predominant influences induced by overtime among Chinese overtime employees based in IT companies and identified potential psychosocial characteristics for mitigating the negative impacts related to overtime. The reported findings add to cumulating evidence of positive effects of reward and work time flexibility on employees’ mental health, fatigue, WFC and job-related outcomes and applicability of ERI and WTC model in overtime situations in IT industry. In order to reduce the adverse experience of overtime, the organization should consider work-related reasons, personal reasons and cultural influence in the workplace. In addition, the model of voluntary and involuntary overtime put forward in this study may help to reduce overtime employees’ mental health complaints and facilitate efficiency, productivity and work engagement.

## Data availability statement

The raw data supporting the conclusions of this article will be made available by the authors, without undue reservation.

## Ethics statement

The studies involving human participants were reviewed and approved by the Ethics Committee of Division of Psychiatry and Applied Psychology, the University of Nottingham. The patients/participants provided their written informed consent to participate in this study.

## Author contributions

JY conceptualized the study, sourced all the materials, facilitated and organized the discussion, analyzed the data, and drafted the manuscript. SL guided the conceptualization of the study, assisted with the facilitation of the discussion, and commented on all drafts to improve quality. All authors have read and agreed to the published version of the manuscript.

## Funding

This research was supported by the Program for the Philosophy and Social Sciences Research of Higher Learning Institutions of Shanxi (no: 2021W091).

## Conflict of interest

The authors declare that the research was conducted in the absence of any commercial or financial relationships that could be construed as a potential conflict of interest.

## Publisher’s note

All claims expressed in this article are solely those of the authors and do not necessarily represent those of their affiliated organizations, or those of the publisher, the editors and the reviewers. Any product that may be evaluated in this article, or claim that may be made by its manufacturer, is not guaranteed or endorsed by the publisher.
